# Clavicle morphometry in the adult Turkish population: implications for fracture management and implant design

**DOI:** 10.1186/s12880-026-02658-9

**Published:** 2026-08-06

**Authors:** Hadi Sasani, Yaşar Mahsut Dinçel, Mazhar Özkan, Gündüz Ercan Kutluay, Murat Erem

**Affiliations:** 1 Department of Radiology, Faculty of Medicine, Tekirdağ Namık Kemal University, Tekirdağ, Türkiye; 2 Department of Orthopedics and Traumatology, Faculty of Medicine, Tekirdağ Namık Kemal University, Tekirdağ, Türkiye; 3 Department of Anatomy, Faculty of Medicine, Tekirdağ Namık Kemal University, Tekirdağ, Türkiye; 4 Department of Orthopedics and Traumatology, Faculty of Medicine, Trakya University, Edirne, Türkiye

**Keywords:** Clavicle, Morphometric analysis, Radiological examination, Orthopaedic implications

## Abstract

**Background:**

This study aimed to evaluate the segmental morphometry of the clavicle using computed tomography (CT) in an adult Turkish population and to assess its potential implications for orthopedic surgical planning and implant design.

**Methods:**

A total of 450 individuals (225 females, 225 males; aged 18–80 years) were retrospectively analyzed. Multiplanar reconstructed CT images were used to define the longitudinal axis of the clavicle. The bone was divided into five equal segments along this axis, and craniocaudal and anteroposterior diameters were measured in each segment. For standardization, only the left clavicle was evaluated. Measurements were performed three times, and mean values were used for analysis. Morphometric parameters were compared according to sex and age groups using appropriate statistical tests, with adjustments for multiple comparisons.

**Results:**

Significant sex-related differences were observed in both craniocaudal and anteroposterior diameters across all clavicular segments (*p* < 0.001). Age-related differences were more pronounced in proximal and midshaft regions, particularly in anteroposterior dimensions. Overall, the clavicle demonstrated segment-dependent morphological variability, with relatively greater dimensions in medial regions compared to lateral segments.

**Conclusions:**

The clavicle exhibits significant segmental and sex-based morphometric variation. These findings may have implications for surgical fixation strategies, particularly in relation to implant positioning, plate contouring, and screw placement. However, clinical interpretations should be considered cautiously, as they are based solely on morphometric data.

## Background

The clavicle, a critical bone in the shoulder girdle, plays a vital role in clinical practice, particularly in the treatment of fractures and the design of fixation devices. Its unique morphology, characterized by distinct regions associated with muscle and ligament attachments, influences both its biomechanical properties and susceptibility to injury [1]. Studies have shown significant gender- and side-specific variations in clavicle geometry, with male clavicles typically being longer, wider, and thicker than females [2]. Advanced methods, such as three-dimensional statistical shape analysis, have provided precise and reproducible measurements, enhancing our understanding of clavicle morphology and informing the development of optimized fixation devices [1].

Clavicle fractures constitute 2.6-4% of adult fractures and 35% around the shoulder. It is anatomically divided into three regions medial, middle, and lateral. Fracture location is approximately 80% in the middle, 15% in the lateral, and 5% in the medial part [3].

While conservative management has traditionally been the standard, recent evidence supports operative treatment for displaced fractures, citing improved functional outcomes and reduced complications [4]. However, the treatment of medial and lateral fractures remains contentious, with intramedullary fixation often favored for its minimally invasive approach [5]. Challenges such as non-union and malunion can often be addressed through surgical interventions, including bone grafting in atrophic cases [6]. Despite ongoing debates, the trend toward operative management for displaced fractures underscores the importance of understanding clavicle anatomy to improve surgical outcomes [4].

The radiological modalities used in the examination of the clavicle are X-ray radiographs and computed tomography (CT) and magnetic resonance imaging are used as cross-sectional methods [7]. CT is used in routine practice because of the advantages of multiplanar reconstruction (MPR) and 3-dimensional imaging as well as internal structure evaluations such as bone density, cortex thickness, and cortical integrity.

This study focuses on clavicle morphometry in the Turkish population, motivated by its clinical relevance in orthopaedics, forensics, and anthropology. Population-specific data are essential, as clavicle measurements can vary significantly across demographics, influencing fracture patterns, implant design, and surgical strategies. Previous research has demonstrated the utility of clavicle morphometry in sex estimation, treatment planning, and joint assessments in Turkish adults [8] emphasizing the need for detailed, localized analyses. This study aims to provide foundational morphometric data using CT images to enhance clinical practice such as implant design in a large adult Turkish population, contributing to improved patient outcomes and advancing orthopaedic practice.

## Methods

The present study is a retrospective cross-sectional study, and a total of 450 patients (225 males, 225 females) aged 18–80 years were included by consecutively screening all thoracic CT examinations performed between 01 January 2023 and 01 December 2024 at the Radiology Department that met the predefined inclusion criteria.

Cases with CT images with optimum image quality, including all parts of the clavicle, were included in the study. Cases affecting the integrity of the clavicle (surgery, trauma, lesions, etc.), cases under 18 years of age, and those over 80 years of age without optimum image quality were excluded from the study. The cases were categorized into age groups (18–40, 41–60, and 61–80 years) according to both sexes in equal numbers (*n* = 75).

The study included CT examinations of 450 participants whose thoracic field of view (FOV) included all parts of the clavicle. A 128-row multi-detector CT scanner (Aquilion™ Prime; Canon Medical Systems) was used for CT scanning. CT acquisition parameters were as follows: current 100–250 mAs modulated by body mass index (BMI) dose; tube voltage 100–140 kV and collimation 0.5 mm x 80, gantry rotation time 0.35 s, pitch factor 0.813, FOV: 20 × 20 cm, slice thickness 1 mm and slice pitch 0.8 mm.

The evaluation and measurements of the patients included in the study were performed by a radiologist with 12 years of experience.

### Morphometric evaluation

**Fig. 1:**
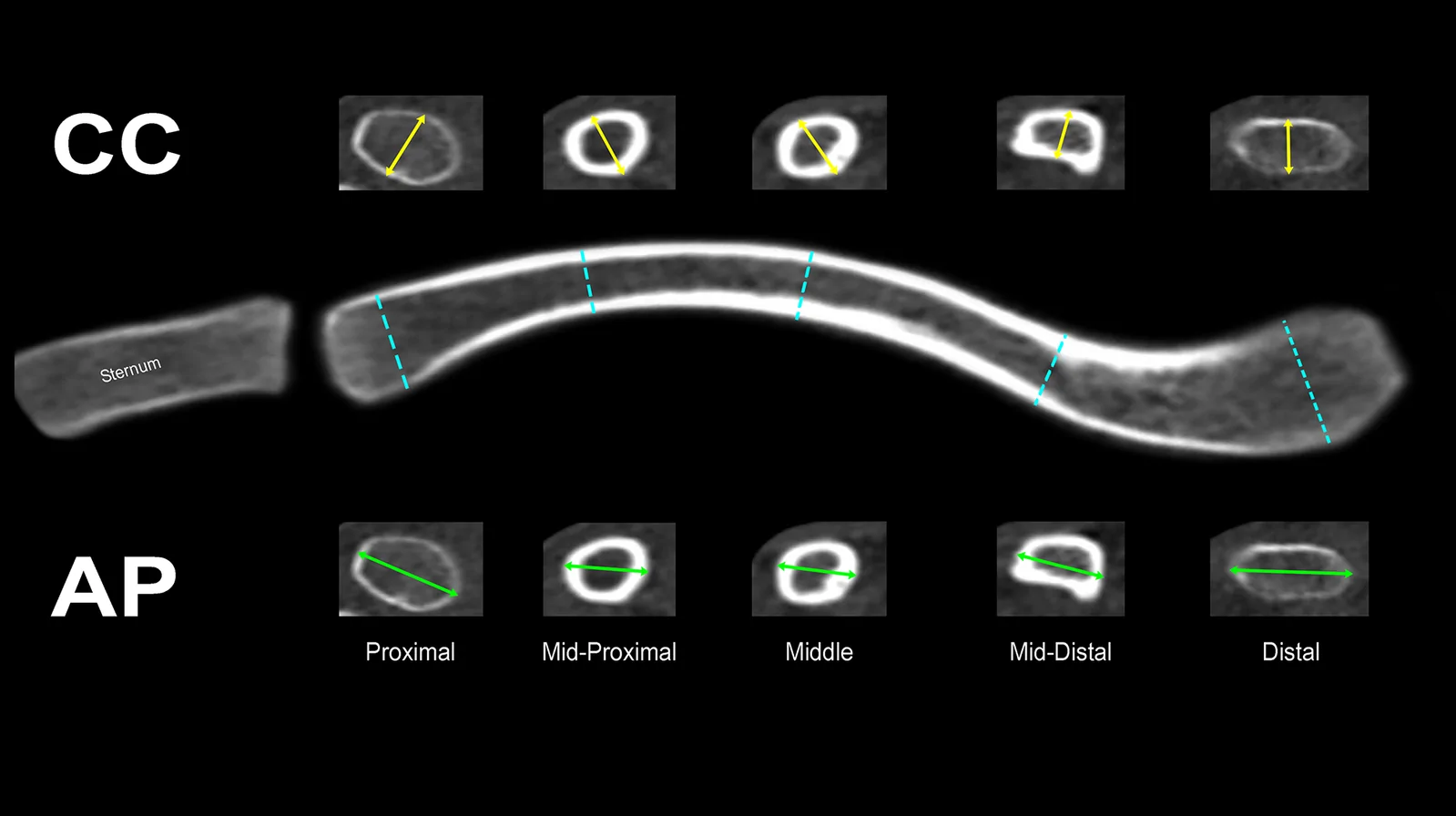
CT image showing antero-posterior and cranio-caudal measurements from different parts of the clavicle

Using multiplanar reconstruction on the Sectra 7.0 workstation (Sectra AB, Linköping, Sweden), a standardized three-axis coordinate system was established for each clavicle. The X-axis represented the mediolateral (long) axis of the clavicle, the Y-axis the anteroposterior dimension, and the Z-axis the craniocaudal dimension. The clavicle was aligned so that its long axis corresponded to the X-axis, and all linear measurements were taken on planes perpendicular to this long axis, ensuring consistent anatomical orientation (Fig. 1).

The total clavicle length was then divided into five equal segments (proximal, mid-proximal, middle, mid-distal, and distal). At each segment, the craniocaudal (CC) and anteroposterior (AP) diameters were measured on cross-sections that were orthogonal to the clavicular long axis. To improve measurement reliability, three consecutive measurements were taken within each segment, and the mean value was used for analysis.

Only left clavicles were evaluated. This decision was made to ensure measurement standardization and methodological consistency, as unilateral assessment eliminates potential variability introduced by side-to-side comparisons. Previous morphometric studies of bilateral symmetric bony structures have similarly adopted a single-side approach to reduce inter-measurement complexity [9]. Although clavicular asymmetry exists, it is generally minor in the absence of handedness-related remodeling or pathology, and the left side was selected to avoid potential dominance-related bias given that the majority of the population is right-handed.

### Statistical analysis

A post-hoc power analysis was conducted using GPower 3.1.9.7 software. The analysis, based on an alpha error probability of 0.05 and an effect size of 0.5, demonstrated a statistical power of 0.99, indicating that the sample size was sufficient to detect significant differences between groups.

Normality tests (Shapiro-Wilk / Kolmogorov-Smirnov) were performed to evaluate whether the data were normally distributed. Proximal, middle, mid-distal, and distal parts of AP measurements showed normal distribution, while CC measurements and AP mid-proximal did not show normal distribution. For normally distributed data, the independent sample T-test was used for comparison of two groups, One-way ANOVA and post-hoc analysis (Tukey’s test) was used for comparison of more than two groups, and Pearson’s test was used for correlation analysis.

For not normally distributed data, the Mann-Whitney U test was used for the comparison of two groups, the Kruskal-Wallis test with Dunn–Bonferroni post-hoc correction was used for the comparison of more than two group, and the Spearman Rho test was used for correlation analysis. The chi-square test was used to analyze categorical data and descriptive tests were used to analyze demographic data.

Receiver Operating Characteristic (ROC) analysis was performed to evaluate the discriminatory power of clavicular measurements for gender estimation. Measurement reliability was assessed using the intraclass correlation coefficient (ICC), which showed excellent agreement (ICC = 0.94). The statistical significance level was accepted as *p* < 0.05.

## Results

The clavicle measurements demonstrated distinct patterns across all five segments. Males exhibited greater AP and CC values than females across all segments, and age-related differences were observed in several regions. Detailed data are presented in Tables 1 and 2.

**Table 1:** Mean ± standard deviation of AP and CC measurements according to gender and age groups and comparison between groups

Parts of the clavicle	Gender (n)	Measurement axis	Age groups (Mean ± SD, mm)		
			18–40	41–60	61–80
Proximal	Male (*n* = 75)	CC	24.56 ± 2.72	26,83 ± 3.01	28,32 ± 3.37
		AP	20.22 ± 2.58^a^	21,96 ± 3.39^b^	22,36 ± 2.59^b^
		*p*	**< 0.001**	**< 0.001**	**< 0.001**
	Female (*n* = 75)	CC	23,40 ± 2.63	24,55 ± 2.34	25,44 ± 2.58
		AP	18,61 ± 1.61^a^	20,38 ± 3.02^b^	21,10 ± 1.92^b^
		*p*	***< 0.001***	***< 0.001***	***< 0.001***
Mid-proximal	Male (*n* = 75)	CC	13.77 ± 2.13	13,36 ± 2.52	13,26 ± 2.44
		AP	13.02 ± 1.71^a^	13,80 ± 2.00^b^	13,98 ± 1.91^b^
		*p*	**< 0.05**	**< 0.05**	**< 0.05**
	Female (*n* = 75)	CC	11,77 ± 2.19	11,93 ± 2.29	12,10 ± 2.41
		AP	11,11 ± 1.76^a^	11,82 ± 1.97^a^	11,66 ± 1.83^a^
		*p*	> 0.05	> 0.05	> 0.05
Middle	Male (*n* = 75)	CC	11.12 ± 1.45	11,22 ± 1.17	11,26 ± 1.38
		AP	12.95 ± 1.58^a^	13,58 ± 1.30^b^	13,61 ± 1.38^b^
		*p*	***< 0.001***	***< 0.001***	***< 0.001***
	Female (*n* = 75)	CC	9,21 ± 0.93	9,40 ± 1.08	9,57 ± 0.90
		AP	10,95 ± 1.22^a^	11,38 ± 1.42^a^	11,76 ± 1.16^a^
		*p*	***< 0.001***	***< 0.001***	***< 0.001***
Mid-distal	Male (*n* = 75)	CC	11.49 ± 1.87	11,31 ± 1.72	10,88 ± 1.64
		AP	15.45 ± 2.28^a^	15,09 ± 2.17^a^	16,21 ± 1.32^b^
		*p*	***< 0.001***	***< 0.001***	***< 0.001***
	Female (*n* = 75)	CC	9,49 ± 1.80	9,41 ± 1.35	9,42 ± 1.15
		AP	12,53 ± 1.78^a^	12,67 ± 1.85^a^	13,65 ± 1.76^b^
		*p*	***< 0.001***	***< 0.001***	***< 0.001***
Distal	Male (*n* = 75)	CC	11.53 ± 2.06	11,82 ± 2.24	12,09 ± 2.66
		AP	20.30 ± 3.78^a^	20,85 ± 3.76^a^	21,78 ± 1.64^b^
		*p*	***< 0.001***	***< 0.001***	***< 0.001***
	Female (*n* = 75)	CC	9,77 ± 1.25	10,01 ± 1.71	10,00 ± 1.33
		AP	17,84 ± 3.35^a^	18,36 ± 3.04^a^	18,94 ± 2.99^a^
		*p*	***< 0.001***	***< 0.001***	***< 0.001***

* Friedman test was used for comparison of repeated measurements in clavicula sections. AP, antero-posterior; CC, craniocaudal. Groups sharing the same superscript letter are not significantly different according to Tukey post-hoc test (*p* < 0.05). Different superscripts indicate significant differences

**Table 2:** Comparison of clavicular CC thickness across age groups (Kruskal–Wallis test and Dunn–Bonferroni post-hoc)

Parameter	Gender	18–40 (Median, Min–Max)	41–60 (Median, Min–Max)	61–80 (Median, Min–Max)	*p* (KW)	Post-hoc Comparisons
CC Proximal (mm)	Male (*n* = 75)	24.00 (21–30)	27.30 (20.40–35)	28.60 (17.40–34)	**< 0.001**	**18–40 < 41–60**; **18–40 < 61–80**; **41–60 < 61–80**
	Female (*n* = 75)	23.30 (17.2–30)	24.60 (19.5–30.8)	25.20 (19.1–24.1)	**< 0.001**	**18–40 < 41–60**; **18–40 < 61–80**; ns: 41–60 vs. 61–80
CC Mid-Proximal (mm)	Male (*n* = 75)	13.50 (9.5–19.6)	12.70 (9.6–20.2)	13.00 (8.2–20.1)	0,158	ns
	Female (*n* = 75)	11.70 (7.6–18.7)	11.70 (7–19.1)	11.50 (8.3–19.3)	0,861	ns
CC Middle (mm)	Male (*n* = 75)	11.00 (7.8–14.4)	11.10 (8.6–14.6)	11.30 (8–15.5)	0,690	ns
	Female (*n* = 75)	9.20 (6.6–8.6)	9.20 (7.2–12.2)	9.60 (7.6–11.6)	0,069	ns
CC Mid-Distal (mm)	Male (*n* = 75)	11.30 (7.6–18.3)	11.30 (8.1–18.6)	10.70 (7.8–16.8)	0,055	ns
	Female (*n* = 75)	9.30 (6.6–14.3)	9.50 (6.3–13.4)	9.40 (6.4–12.5)	0,801	ns
CC Distal (mm)	Male (*n* = 75)	11.20 (7.3–22.2)	11.50 (8.1–26.3)	11.70 (7.5–23.5)	0,403	ns
	Female (*n* = 75)	9.80 (7.1–12.8)	10.0 (6.8–19.4)	10.0 (6.5–13.5)	0,502	ns

KW: Kruskal–Wallis ANOVA. Post-hoc: Dunn–Bonferroni test. ns: non-significant (*p* > 0.05). Bold p-values indicate statistical significance

### Analysis by gender

**Fig. 2:**
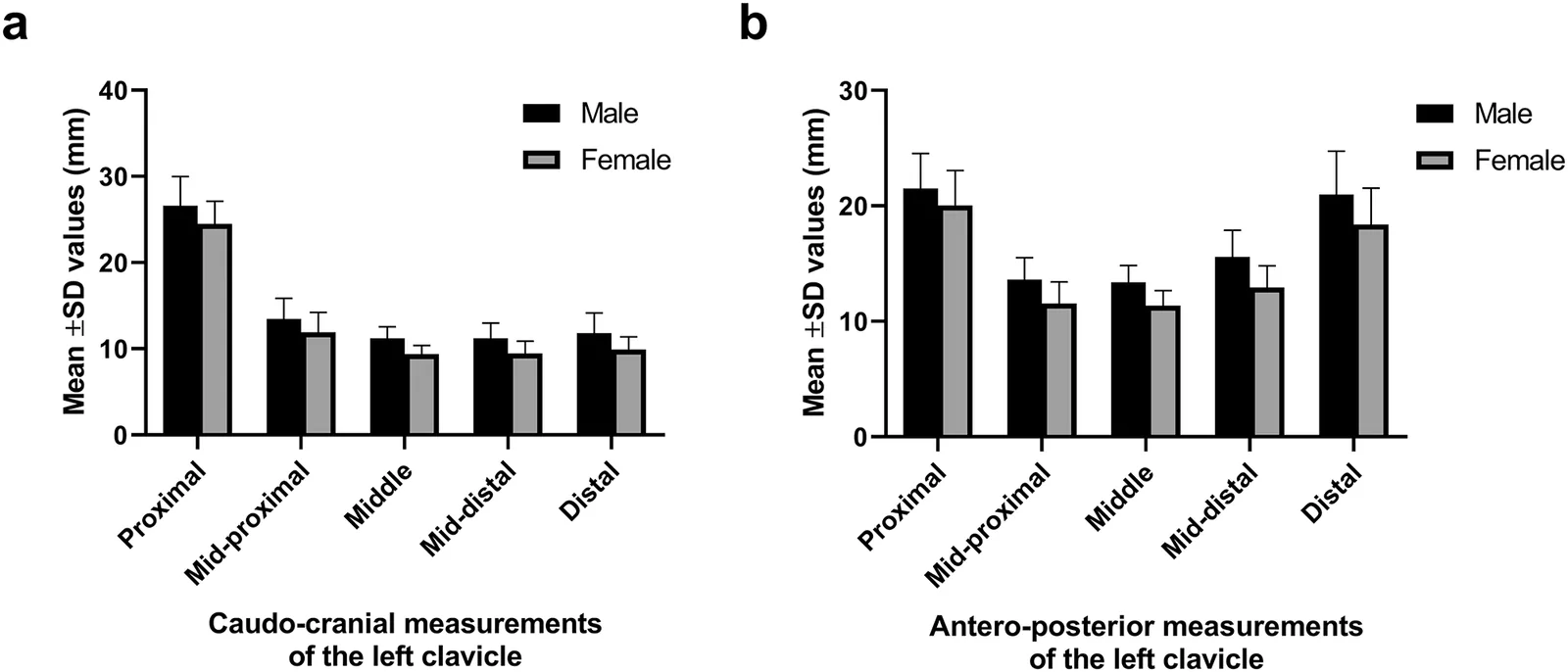
Comparison of measurements with clavicle in men and women. **a**, cranio-caudal measurements; **b**, antero-posterior measurements of the left clavicle

All CC and AP measurements (proximal, mid-proximal, middle, mid-distal, and distal) showed statistically significant inter-gender differences (*p* < 0.001), indicating consistent sexual dimorphism throughout the entire length of the clavicle (Table 1; Fig. 2). Male values were higher than female values across all age groups and all segments (*p* < 0.05). The proximal segment demonstrated the greatest absolute difference between sexes, suggesting it may carry particular relevance for gender-based implant sizing and forensic morphometric analysis.

### Analysis by age groups

Participants were categorized into three groups — young (18–40), middle-aged (41–60), and elderly (61–80) — reflecting established epidemiological intervals and the World Health Organization’s definition of older populations [10]. Statistically significant age-related differences were identified in several parameters (Tables 1 and 2); key findings are summarized below.

CC proximal showed significant differences across all three age group comparisons (*p* < 0.001), representing the most age-sensitive parameter in this dataset. Values increased progressively with age in both sexes (Table 2), suggesting that proximal clavicular thickening may reflect cumulative periosteal remodeling and could have implications for medial clavicle implant design and surgical planning.

AP measurements showed more localized age effects: AP proximal and AP mid-proximal differed significantly between young and both older groups (*p* < 0.001 and *p* < 0.05, respectively), while AP mid-distal and AP distal showed significant differences only involving the elderly group (*p* < 0.05). These findings suggest that age-related morphological change is more pronounced in the proximal clavicle, while the distal segment remains relatively stable until later in life.

### Correlation analysis

**Fig. 3:**
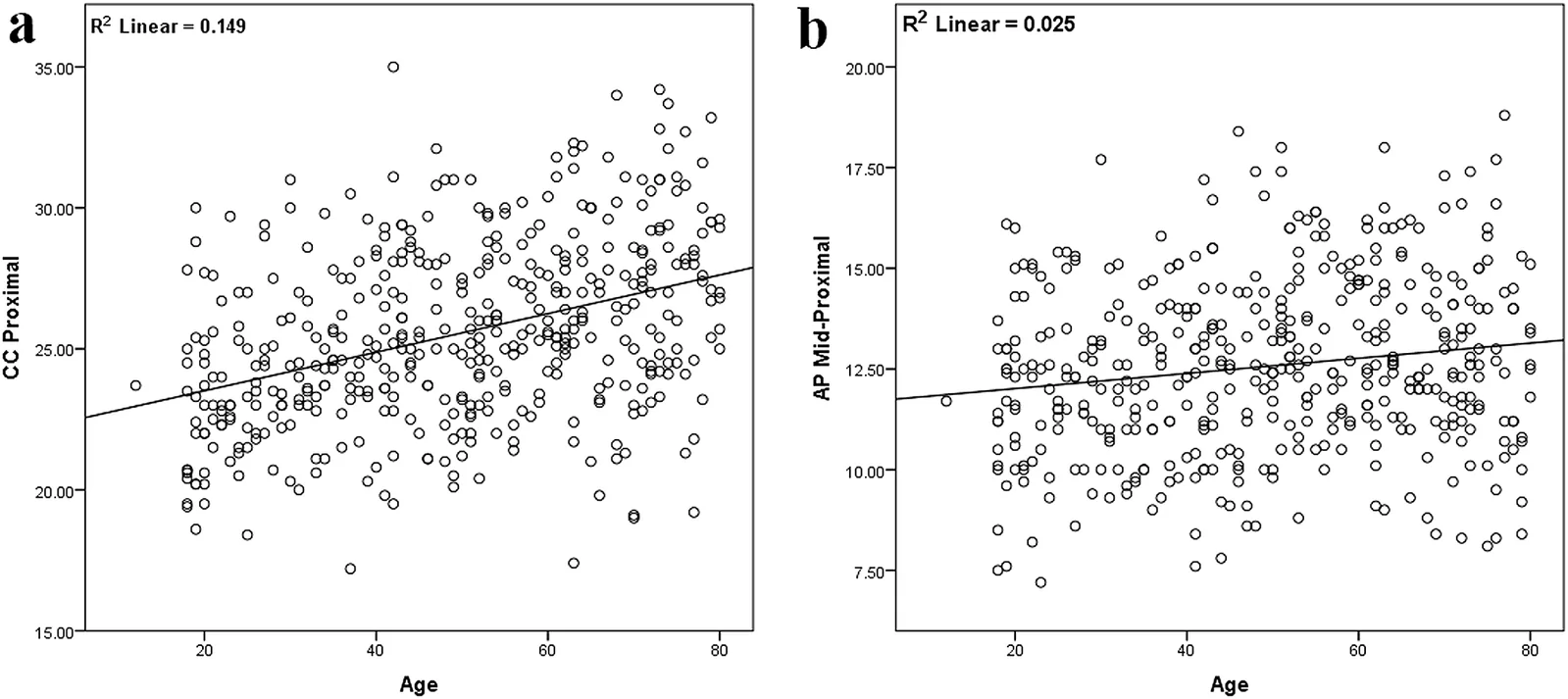
Correlation graphs of two parameters related to age. **a**, proximal measurement value of the craniocaudal part of the left clavicle; **b**, mid-proximal measurement value of antero-posterior part of the left clavicle

Pearson’s and Spearman’s correlation analyses confirmed positive associations between age and multiple morphometric parameters: CC proximal (*r* = 0.392), AP proximal (*r* = 0.348), AP mid-proximal (*r* = 0.149), AP middle (*r* = 0.186), AP mid-distal (*r* = 0.166), and AP distal (*r* = 0.141), all reaching statistical significance (*p* < 0.05). CC proximal showed the strongest correlation with age, reinforcing its value as a morphometric indicator of age-related change (Fig. 3a–b).

### Gender estimation (ROC and discriminant analysis)

**Fig. 4:**
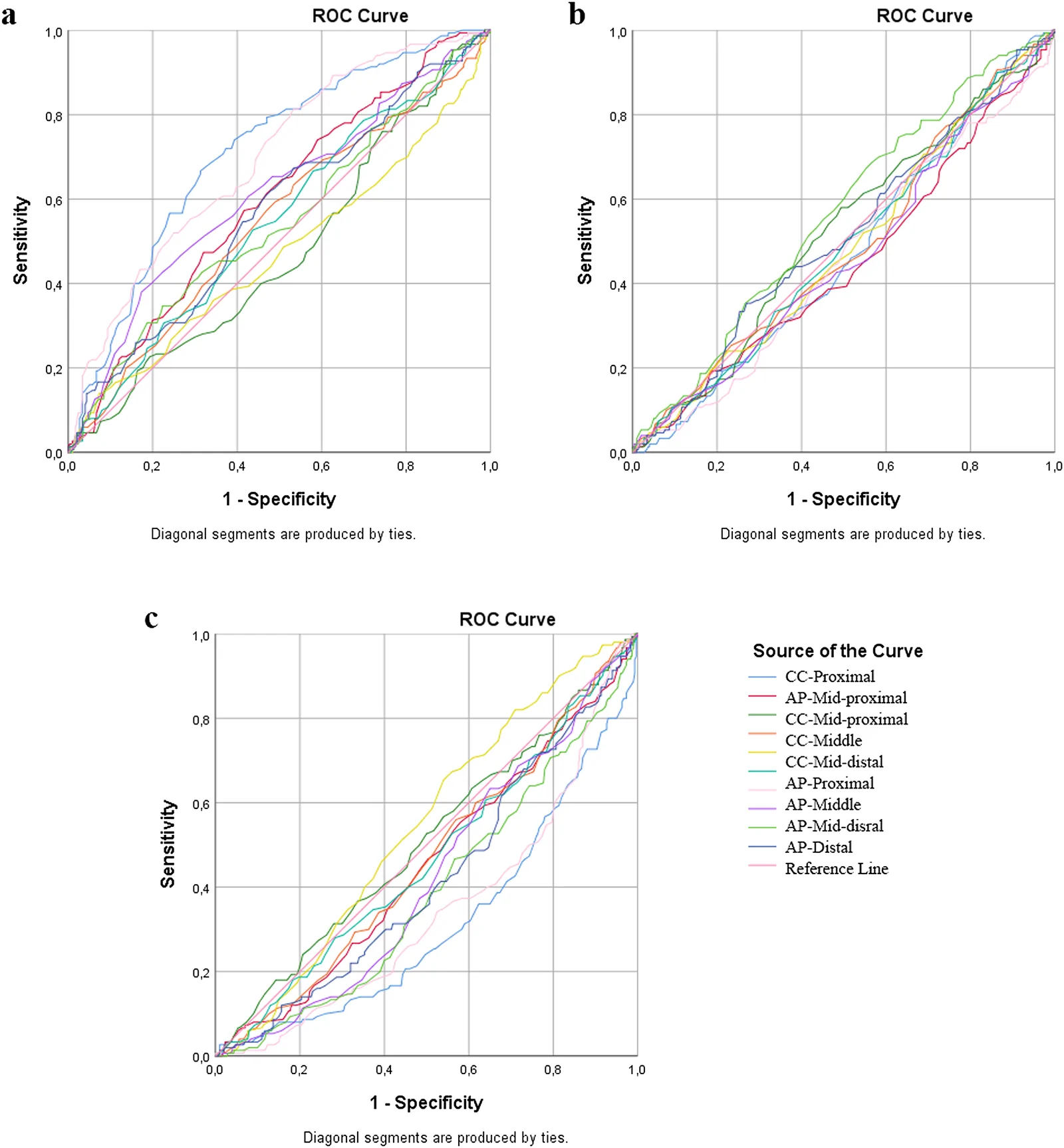
ROC curve analysis results in different age groups. **a**, 18–40 years; **b**, 41–60 years and **c**, 61–80 years old

ROC and stepwise discriminant analyses were performed to evaluate the utility of clavicular measurements in gender determination. AUC values, sensitivity, specificity, and optimal cut-off points are reported in Tables 3 and 4. ROC curves for the most discriminating parameters across age groups are illustrated in Fig. 4.

**Table 3:** Receiver operating characteristics curve analysis results for sex determination in different age groups

Groups	Variable	Cut-off	*p*- value	AUC (95% CI)	Sensitivity	Specifity
18–40 years	CC-prox	24,9	< 0,001	,288 (,239-,338)	68	67
	AP-mid-prox	12,35	0,001	,402 (,348-,457)	57	58
	AP-prox	20,75	< 0,001	,305 (,254-,355)	66	56
	AP-middle	12,15	< 0,001	,389 (,333-,446)	58	59
	AP-distal	19,25	0,018	,432 (,375-,488)	55	56
41–60 years	AP-mid-distal	14,15	0,026	,564 (,509-,620)	55	55
61–80 years	CC-prox	25,55	< 0,001	,316(,264-,368)	36	37
	AP-prox	20,90	< 0,001	,346(,293-,398)	37	40
	AP-middle	12,45	0,009	,424(,370-,478)	47	46
	AP-mid-distal	14,25	< 0,001	,390(,335-,444)	43	45
	AP-distal	19,75	0,006	,420(,365-,475)	43	43

* The abbreviations used in the table are as follows: AP, antero-posterior; CC, craniocaudal; AUC, area under the curve; CI, confidence interval; prox, proximal

**Table 4:** Discriminant analysis results for gender determination

Variables	Canonical discriminant function coefficients		Wilks’ Lambda	Functions at group centroids	Accuracy
	Unstandardized	Standardized			
CC-Middle	,420	,492	,481	Male= 1,037	85,3%
CC-Distal	,132	,257			
AP-Proximal	-,059	-,179			
AP-Mid-Proximal	,127	,240			
AP-Middle	,219	,303		Female= -1,037	
AP-Mid-Distal	,170	,356			
Constant	-11,273	-			

## Discussion

The present study provides a comprehensive segmental morphometric analysis of the left clavicle using CT imaging in a large, sex- and age-balanced adult population. The principal findings were threefold: (1) clavicular dimensions were greatest in the proximal and midshaft segments and progressively narrowed toward the distal end; (2) significant sexual dimorphism was present across all segments, with males demonstrating greater CC and AP values; and (3) age-related morphological change was most pronounced in the proximal segment, particularly CC proximal, which showed the strongest correlation with age (*r* = 0.392).

### Regional morphometry and comparison with previous studies

The segmental distribution observed in the present study — with larger dimensions proximally and progressively narrower dimensions distally — is consistent with prior morphometric investigations [11–13]. Yarar et al. [14] and Ishwarkumar et al. [13] reported comparable patterns of regional thickness variation and sex-related differences in dry bone specimens (Table 5). However, several specific measurements, particularly at the acromial end, differed from our CT-based findings. These discrepancies are likely attributable to population variability, methodological differences, and the inherent limitations of dry bone specimens, in which postmortem dehydration and environmental exposure may alter bone dimensions [15, 16]. CT-based in vivo measurements, as used in the present study, avoid these confounders and may therefore provide more clinically applicable reference data. Notably, our sample also includes age and sex stratification absent from many dry bone studies, enhancing generalizability.

**Table 5:** Summary of the literature and comparison with our findings

Authors (Year)	Population (Country)	Method (Sample)	Findings (Mean ± SD, mm)				
			Proximal (Sternal)	Mid-proximal	Middle	Mid-distal	Distal (Acromial)
Walters et al. (2010)	South Africa	Dry Bones (*n* = 100, both)	AP:18 CC:14	AP:13,6 CC:10	AP:14 CC:10	AP:19.3 CC:11	AP:23 CC:11
Yarar et al. (2024)	Turkish	Dry Bones (*n* = 46, left)	AP: 20.3 ± 4.1 CC: 23.3 ± 4.1	na	AP: 12.1 ± 1.5 CC: 9.9 ± 1.3	na	AP: 26.1 ± 4.7 CC: 10.8 ± 1.7
Bozdag et al. (2020)	Turkish	CT images (*n* = 152, left)	AP: na CC: 22.4 ± 2.5	na	AP: 10.5 ± 1.1 CC: na	na	AP: 22.7 ± 2.9 CC: na
Our study	Turkish	CT images (*n* = 450, left)	AP: 20.8 ± 3.1 CC: 25.5 ± 3.2	AP: 12.6 ± 2.2 CC: 12.7 ± 2.5	AP: 12.4 ± 1.7 CC: 10.3 ± 1.5	AP: 14.3 ± 2.5 CC: 10.3 ± 1.8	AP: 19.7 ± 3.7 CC: 10.9 ± 2.2

* The abbreviations used in the table are as follows: AP, antero-posterior; CC, craniocaudal; na, not applicable; SD, standard deviation

### Sexual dimorphism

Males demonstrated significantly greater CC and AP dimensions across all five segments (*p* < 0.001), confirming robust sexual dimorphism throughout the entire clavicle. This is consistent with prior reports [8, 11, 17] and carries direct clinical relevance. Differences in bone thickness and geometry between sexes influence plate contouring, screw length selection, and implant sizing. Furthermore, sex-related variation in clavicle-acromion relationships has been associated with subacromial erosion following fixation procedures [18]. Our findings support the need for sex-specific morphometric reference data in surgical planning, and suggest that implant systems designed without sex stratification may not adequately accommodate the anatomical range of the population.

### Age-related changes

CC proximal was the parameter most strongly associated with age (*r* = 0.392, *p* < 0.05), with mean values increasing progressively from young to elderly groups in both sexes. AP proximal and AP mid-proximal also showed significant age-related differences, though with weaker correlations. In contrast, distal segment measurements remained relatively stable until the elderly group, suggesting that age-related morphological change follows a proximal-to-distal gradient. While periosteal remodeling with aging may partly explain these observations [19–22], our study did not include bone mineral density or biomechanical data, and the underlying mechanisms cannot be directly established from morphometric measurements alone. Future studies combining CT morphometry with bone quality assessments would help clarify whether the proximal changes reflect adaptive remodeling or pathological bone loss.

### Clinical implications

The segmental data generated by this study have several direct clinical applications. Clavicle fractures most frequently occur in the midshaft, which in our study demonstrated relatively larger dimensions — yet this region’s biomechanical characteristics and susceptibility to fracture remain multifactorial [23–28]. Distal segment fractures present particular technical challenges owing to the narrow bone dimensions we observed, which limit fixation options and complicate implant placement [29, 30]. Our morphometric data quantify this narrowing and may help guide distal implant design for the Turkish population.

Regarding screw placement, the CC and AP dimensions measured at each segment provide direct reference values for safe screw length determination. Excessive screw penetration carries risk of neurovascular injury, including pseudoaneurysm and arteriovenous fistula [31, 32], and prior studies have documented neurovascular contact in a significant proportion of fixation cases [33]. The segment-specific thickness values reported here may help surgeons select appropriate screw lengths and reduce this risk, particularly in the proximal and midshaft regions where neurovascular proximity is greatest.

Finally, population-specific morphometric data are increasingly recognized as essential for implant compatibility, given that clavicular dimensions vary across ethnicities [9, 11, 34]. The present study contributes Turkish population reference data, which may improve the fit of locally used implant systems and inform future population-tailored device development.

### Limitations

This study has several limitations. First, only the left clavicle was evaluated. While this decision was made to ensure methodological consistency and avoid inter-side variability, it precludes assessment of bilateral asymmetry, which may itself carry morphometric significance. Second, all measurements were performed by a single observer; although intraobserver reliability was excellent (ICC = 0.94), interobserver variability was not assessed. Third, clinical parameters including BMI and bone mineral density were not incorporated, limiting mechanistic interpretation of the age-related findings. Fourth, analysis was restricted to linear measurements; three-dimensional characteristics such as curvature, torsion, and surface geometry — which are relevant to both biomechanical modeling and implant contouring — were not evaluated. Fifth, the power analysis was conducted post-hoc rather than a priori, which represents a methodological limitation; although the analysis confirmed adequate statistical power (0.99), prospective sample size calculation would have provided a stronger design rationale. Future studies incorporating bilateral analysis, multi-observer validation, 3D imaging, and bone quality parameters would provide a more complete understanding of clavicular morphology and its surgical implications.

## Conclusions

This study provides comprehensive CT-based morphometric data on the clavicle in a large adult population, demonstrating significant segmental, sex-related, and age-related variability. The findings indicate that clavicular dimensions are not uniform along the bone, with relatively greater measurements in proximal and midshaft regions and smaller dimensions toward the distal segment.

These variations may have implications for surgical planning, particularly in relation to implant positioning, plate contouring, and screw selection. However, such clinical considerations should be interpreted cautiously, as the present study is based solely on morphometric analysis.

The results also highlight the importance of considering population-specific anatomical differences in the design and selection of fixation devices. Incorporating detailed morphometric data into surgical planning and implant development may contribute to more individualized approaches and potentially improved clinical outcomes. Future studies incorporating bilateral analysis, three-dimensional imaging, and bone quality parameters would further strengthen the clinical applicability of these findings.

## Data Availability

The datasets used and/or analysed during the current study are available from the corresponding author on reasonable request.

## Declarations

### Declarations

This manuscript is an original work, has not been published previously either in whole or in part, and is not under consideration for publication by any other journal. All data, tables, figures, etc. used in the manuscript were prepared originally by the authors.

## Abbreviations

AP: Anteroposterior
AUC: Area under the curve
BMI: Body mass index
CC: Craniocaudal
CT: Computed tomography
ICC: Intraclass correlation coefficient
MPR: Multiplanar reconstruction
ROC: Receiver operating characteristic
